# The Role of Butylidenephthalide in Targeting the Microenvironment Which Contributes to Liver Fibrosis Amelioration

**DOI:** 10.3389/fphar.2016.00112

**Published:** 2016-04-27

**Authors:** Hong-Meng Chuang, Hong-Lin Su, Chien Li, Shinn-Zong Lin, Ssu-Yin Yen, Mao-Hsuan Huang, Li-Ing Ho, Tzyy-Wen Chiou, Horng-Jyh Harn

**Affiliations:** ^1^Agricultural Biotechnology Center, Department of Life Sciences, National Chung Hsing University Taichung, Taiwan; ^2^Department of Life Science and Graduate Institute of Biotechnology, National Dong Hwa University Hualien, Taiwan; ^3^Center for Neuropsychiatry, China Medical University Hospital Taichung, Taiwan; ^4^Graduate Institute of Immunology, China Medical University Taichung, Taiwan; ^5^Division of Respiratory Therapy, Department of Chest Medicine, Taipei Veterans General Hospital Taipei, Taiwan; ^6^Department of Pathology, China Medical University Hospital Taichung, Taiwan; ^7^Department of Medicine, China Medical University Taichung, Taiwan

**Keywords:** liver fibrosis, epithelial-messenchymal transition, hepatic stellate cells, regeneration, microenvironment

## Abstract

The treatment of liver fibrosis has clinical limitations because of its multiple etiologies, such as epithelial–mesenchymal transition (EMT) promotion, cell regeneration and remodeling dysfunction, inflammatory cell activation, and scar tissue deposition. These factors might be considered as a new target for the fibrotic microenvironment, leading to increased fibrogenesis and liver fibrosis. Here, we investigate a small molecule named butylidenephthalide (BP) and its multiple effects on liver fibrosis treatment. Thioacetamide was used *in vivo* to induce chronic liver fibrosis. BP was administered orally in rats for a period of 2 and 4 weeks, which resulted in a significantly reduced fibrosis score (*p* < 0.05) and (*p* < 0.001), respectively. The inflammatory reaction of macrophage infiltration were reduced in the administration of BP, which led to the decrease in the transaminase levels. Moreover, we also found liver functions recovering (due to the increased serum albumin and reduced prothrombin time) where liver cells regenerated, which can be seen in the increase of Ki-67 on Oval cell. In addition, the fibrotic scar was also reduced, along with the expression of matrix metalloprotease by hepatic stellate cell. Furthermore, regarding the mechanism/study of EMT reduced by BP, the knockdown of BMP-7, which could reduce α-SMA expression, was mediated by the regulation of TGF-β, which implies its major role on EMT. Finally, in the *in vivo* study, BP treatment of liver fibrosis was reduced by *Bmp7* knockdown in zebrafish, suggesting that BP leads to the reduction of liver fibrosis, which also depends on BMP-7 induction. These results suggest that BP had multiple targets for treating liver fibrosis in the following ways: reduction of EMT, decreasing inflammatory reaction, and liver cell proliferation. This multiple targets approach provided a new mechanism to treat liver injury and fibrosis.

## Introduction

The liver fibrosis occurs when there is an imbalanced state characterized by increased fibrogenesis and decreased fibrinolysis (Gunther et al., 1999), which results in the accumulation of ECM proteins (e.g., collagens, fibronectin; Kendall and Feghali-Bostwick, 2014). The recruitment of myofibroblasts (Knittel et al., 1999a) and immune cells (Bonacchi et al., 2003) by HSCs steadily increase the fibrosis effect. The activated HSCs transdifferentiate into myofibroblast-like cells through epithelial–mesenchymal transition (EMT) (Choi and Diehl, 2009; Fan et al., 2015) stimulates the secretion of transforming growth factor β (TGF-β), which is the primary source of ECM modulator (Liu et al., 2006). Bone morphogenetic protein 7 (BMP-7) has been reported to have the opposite function of TGF-β (Zeisberg et al., 2003; Weiskirchen et al., 2009); in addition, it antagonizes the pathways of TGF-β so that it can have a therapeutic potential (Henderson et al., 2013). Also, since the liver is regenerative, the hepatic oval/progenitor cell differentiates into the hepatocyte (Hindley et al., 2014) and restore its function. Meanwhile, the regenerative cells can replace impaired tissues and also secret matrix metalloproteinases (Duarte et al., 2015) in order to digest scar tissue. These effects seem to be essential in hepatic restoration.

Butylidenephthalide (BP) is abundant in Chinese medicinal herbs such as *Angelica sinensis* (Lao et al., 2004), and *Ligusticum chuanxiong* (Chan et al., 2009). Studies have reported that phthalide compounds perform an antiproliferative function in HSCs through the inhibition of platelet-derived growth factor (PDGF) (Lee et al., 2007), but in the *in vivo* data on fibrosis, this has not yet been reported. In our previous studies, BP was examined for antihepatocellular carcinoma activity by inducing Nur77 (also known as NR4A1) expression, which led to caspase-3-dependent apoptosis (Harn et al., 2011) and triggered an antiplatelet effect through PDGF reduction (Liu et al., 2011). BP also downregulates EMT-related genes such as Snail (Snail/SNAI1) and Slug (Slug/SNAI2) (Yen et al., 2015). Although EMT and tumor migration are the characteristics of activated HSCs that cause liver fibrosis, these considerations prompted us to study the effects of BP on liver fibrosis.

Although the combination approach may solve the problem in the current use of drugs (Trautwein et al., 2015), adequate targeting designation is still a problem. One of the clinically used drugs, Pentoxyfilline, was proven to have beneficial effects on liver fibrosis (Satapathy et al., 2007). However, the improvement in fibrosis was not statistically significant in patients (Zein et al., 2011). In the current study, we found BP has significantly shown improvement in reducing liver fibrosis compared to Pentoxyfilline. In addition, BP has a multi-function capacity that reduces EMT, decreases hepatic inflammation, regenerates hepatic cells, and secretes matrix metalloproteinase. These effects might be considered a new target for the fibrotic microenvironment, and might be ineffective with current drugs.

## Materials and Methods

### Isolation of Hepatic Stellate Cells

The HSCs were isolated using the method described by Kawada et al. (1993) with slight modification. In brief, the Wistar rats were sacrificed, and then perfusion through portal vein with Hank’s balanced salt solution (HBSS) was carried out. The liver was cut off and minced rapidly, and incubated at 37°C for 30 min with constant shaking under 0.05% collagenase I HBSS. The digested liver was filtered through a 100-μm filter and gauze and centrifuged at 50 × *g* for 5 min; the supernatant was then collected and pelleted at 450 × *g* for 10 min. Density centrifugation was performed at 1700 × *g* for 15 min by an equal volume of the suspension and 16.8% Histodenz (Sigma–Aldrich). The HSCs were collected and washed with HBSS three times and then seeded on a culture dish (1 × 10^7^ cells). The cells were cultured in DMEM containing 10% FBS and the medium was replaced every other day for 2 weeks. Our animal studies were approved by the China Medical University Institutional Animal Care and Use Committee.

### Cell Lines and Compound

The HSC-T6 cell line was kindly provided by Professor Friedman of the Mount Sinai School of Medicine (New York, NY, USA). Primary rat HSCs and HSC-T6 cells were maintained in DMEM containing 10% FBS, 1% HEPES, 1% sodium pyruvate, 1% sodium bicarbonate, and 100 ng/mL of penicillin and streptomycin. The cell culture media and supplements were purchased from Thermo Scientific Hyclone (Logan, UT, USA). The cells were cultured at 37°C by using a 5% CO_2_ incubator. BP (CAS 551-08-6) (MW = 188.23) was purchased from Lancaster Synthesis Ltd. (Newgate Morecambe, UK) and dissolved in dimethyl sulfoxide (DMSO) for cell culture. For the animal studies, BP was dissolved in olive oil for oral administration.

### Knockdown through siRNA Transfection

BMP-7 siRNA was synthesized by Thermo Scientific Dharmacon (Lafayette, CO, USA). The TurboFect siRNA transfection reagent was purchased from Fermentas Inc. (Glen Burnie, MD, USA). To ensure that transfection was accurate, we used a pTY-EGFP lentiviral vector carrying the EGFP reporter gene as a positive control, and used 30 and 60 pmol of BMP-7 siRNA in each transfection.

### RNA Extraction and qRT-PCR Analysis

To isolate the RNA, the RNeasy RNA isolation kit was purchased from QIAGEN (Valencia, CA, USA), and used according to the manufacturer’s instruction. Complimentary DNA (cDNA) was synthesized through reverse transcription of 1 μg of total RNA. Primers were synthesized by Genomics BioSci & Tech (Taipei, Taiwan) and are listed below. *Bmp7* forward: 5′-GGTCGGCAGGACTGGATCAT-3′, and reverse: 5′-ACCAGTGTCTGGACGATAGC-3′; *acta* forward:5′-CCGAGATCTCACCGACTACC-3′, and reverse: 5′-TCCAGAGCGACATAGCACAG-3′; *TGFb* forward: 5′-TGACGTCACTGGAGTTGTCCGGCAG-3′, and reverse: 5′-GGGCTTGCGACCCACGTAGTAGACA-3′; *gapdh* forward: 5′-AGCCCAGAACATCATCCCTG-3′; and reverse: 5′-CACCACCTTCTTGATGTCATC-3′. The parameters were established by denaturing the cells at 95°C for 1 min, annealing the cells at 56°C for 1 min, and executing extension at 72°C for 2 min. The PCR products were separated on 2% agarose gels and stained with ethidium bromide. For quantitative RT-PCR, we used 50 ng RNA synthesized cDNA for each reaction by Invitrogene SYBR^®^ GreenER^TM^ qRT-PCR Kit (Logan, UT, USA) and analyzed it on StepOnePlus Real-Time PCR system (Applied Biosystems) or in 2% agarose gel followed by Ethidium bromide staining.

### Western Blot Analysis

Cells were lysed with PRO-PREP (iNtRON Biotechnology, Gyeonggi-do, Korea) and incubated on ice for 15 min. The chilled cells were centrifuged at 15 000 × *g* for 5 min, and the supernatant was then quantified and electrophoresis performed. After the proteins were transferred onto a polyvinylidene fluoride membrane, blocking was then performed using 5% skim milk. Primary antibodics against BMP-7 (orb100464) was purchased from Biorbyt. Antibodies against TGF-β (GTX110630), and collagen I (GTX20292) were purchased from Genetex. Antibody against slug (9585) was purchased from Cell Signaling Technology, and p-Smad 2/3 (sc-11769) was purchased from Santa Cruz. These antibodies were 1000X diluted into the blocking reagent and reacted overnight. The membranes were washed three times with 0.1% Tween 20 in phosphate-buffered saline (PBS; PBST) and incubated with diluted secondary antibodies. All proteins were detected using the Western Lightning Plus ECL reagent.

### Establishment of the Rat Model of Chronic Liver Fibrosis

To establish the rat model for chronic liver fibrosis, 200 mg/kg of TAA (Sigma–Aldrich) was injected intraperitoneally into 8-week-old male Wistar rats every 3 days, and normal saline was injected simultaneously as a control. The therapeutic control entailed using the pentoxifylline (50 mg/kg) to treat TAA-induced rats. The rats were sacrificed on days 30, 45, and 60 for analysis (*n* = 4); cardiac blood samples were collected to measure the biochemical liver function index; liver samples were obtained, processed, and sectioned to evaluate the histopathologic score. Histopathology analysis was conducted as previously described by using H&E and Masson’s trichrome stain (Harn et al., 2012). Liver tissue samples were fixed for 2 days and fixation was in 10% buffered formalin. The tissues were sectioned serially 4-μm of the processed tissue and stained with H&E and Masson’s trichrome stain.

### Establishment of the Fish Model of Chronic Liver Fibrosis

Zebrafish were maintained according to the established protocols of the Zebrafish International Resource Center. Embryos, larvae, and adult fish were maintained in a tank system controlled at 28°C and supplied with a continuous flow of water, and underwent an automated 14-h light and 10-h darkness cycle. The feeding supplies included appropriate kind of food and frequency which was according to the regular care and maintenance protocol (Avdesh et al., 2012). To establish liver fibrosis, as described by Rekha et al. (2008), we used 2-month-old zebrafish and applied 300 mg/kg TAA intraperitoneal injections three times per week for 4 weeks.

### *In Vivo* Knockdown Using Morpholino in Zebrafish

Vivo-MO is an siRNA conjugated with Morpholino specifically targeting BMP-7 for *in vivo* studies, which was purchased from Gene Tools, LLC (Philomath, OR, USA). To perform BMP-7 knockdown, MO was intravenously injected using a retro-orbital injection technique (Pugach et al., 2009). Each fish was placed in dorsal recumbency, on a wet sponge and swimming in tricaine solution (4 mg/mL) to anesthetize the fish. The Hamilton syringe was used to inject through the fish eyes and 5 μL was injected for each fish.

### Histopathological Analysis

For histopathological analysis, liver tissue samples were fixed in 3.7% formaldehyde for 2 days. The tissues were then dehydrated, cleared, and infiltrated by automatic histoprocessor (Tissue-Tek; Sakura, Tokyo, Japan) for 16 h. Serial 4-μm sections of the processed tissue were stained with H&E and Masson’s trichrome stain. For Masson’s trichrome staining, sections were immersed in Bouin’s solution at 56°C for 1 h and then stained in Mayer’s hematoxylin solution for 5 min, in Biebrich scarlet–acid fuchsin solution for 15 min, in phosphomolybdic acid–phosphotungstic acid for 15 min, and in aniline blue for 5 min (all reagents were purchased from Sigma–Aldrich, Steinheim, Germany). The samples were dried and mounted on glass slides and the sections were examined using a microscope (IX70; Olympus Tokyo, Japan). The Metavir Scoring was evaluated by a pathologist, as previously described (Harn et al., 2012). In brief, the Metavir Score for liver fibrosis, were assigned to two standardized numbers to grade on a 5-point scale for fibrosis (F0∼F4) and 4-point scale for the amount of inflammation (A0∼A3).

### Immunohistochemical Staining

Paraffin sections were dehydrated in an oven before they were stained, and were deparaffinized in xylene and rehydrated through a graded series of ethanol solutions. Tissues were blocked with a solution containing 5% milk and 1% bovine serum albumin in PBS for 60 min at room temperature, and subsequently incubated at 4°C overnight with anti-TGF-β, -BMP-7, -αSMA, and -Ki-67 antibodies (GeneTex, USA). Secondary antibodies were hybridized, and visualized by 0.5 mg/mL of diaminobenzidine (DAB). Finally, the sections were counterstained with hematoxylin and mounted, and each tissue samples were randomly photographed at a magnification of 200× by using an Aperio scanner (Leica Biosystems).

### Zymography Analysis

MMPs activity were obtained by zymography analysis. The supernatant was collected after 24 h treatment. The samples buffered without 2-Mercaptoethanol and separated by electrophoresis in a polyacrylamide gel containing 0.2% gelatin (Sigma–Aldrich Co.) The gel was washed with washing buffer (40 mM Tris-HCl pH 8.5, 0.2 M NaCl, 10 mM CaCl2, 2.5% Triton X-100) twice for 30 min. The gel was incubated in the reaction buffer (40 mM Tris-HCl pH 8.5, 0.2 M NaCl, 10 mM CaCl2, 0.01% NaN3) over 16 h. The gel was then stained by Coomassie blue (0.2% Coomassie blue R-250, 50% methanol, 10% acetic acid).

### Statistical Analysis

In the RT-PCR analysis, quantification was conducted using ImageJ (NIH, USA). The IHC positive stained areas were measured using Image Scope (Leica Biosystems). Multiple groups were subjected to a student *t*-test, and *P* ≤ 0.05 indicated the statistical significance.

## Results

### Butylidenephthalide Reduces Liver Fibrosis in Thioacetamide-Treated Rats

**FIGURE 1:**
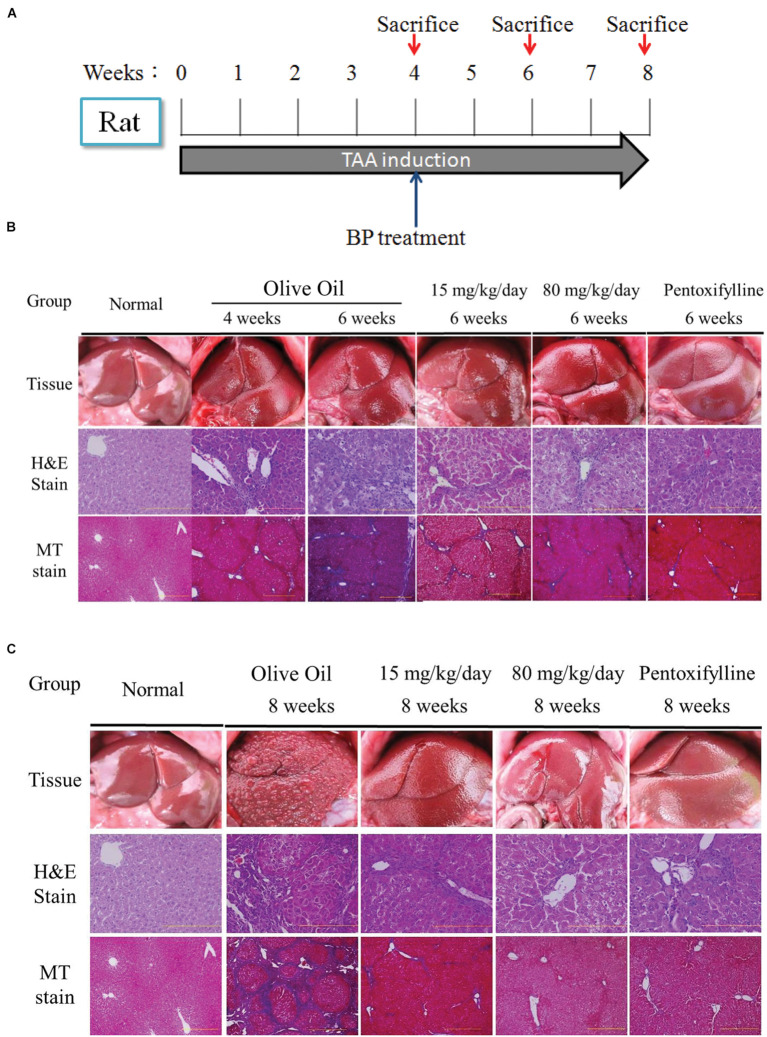
**Butylidenephthalide (BP) ameliorates hepatic fibrosis induced by TAA.** Chronic liver fibrosis model and BP treatment for 2 months. Olive oil treatment was performed as a vehicle treatment group, and the Pentoxfylline treatment (50 mg/kg) was the therapeutic group. Rats were sacrificed at 6th and 8th week for evaluation of liver fibrosis. The Wistar rat were sacrificed on 4th, 6th, 8th weeks. Liver tissue was collected and indicated by H&E and Masson’s trichrome stain. **(A)** Experimental protocol for liver fibrosis model and sacrifice points. Histology on **(B)** 6th week and **(C)** 8th week. The right side indicates arbitrary units by using Aperio ePathology Software (*n* = 5 rat for each group; Original magnification was 100×).

To evaluate the antifibrosis effect of BP, we established a rat model of liver fibrosis by intraperitoneally injecting the rats with TAA (200 mg/kg body weight) twice per week for 8 weeks. Four weeks after TAA injection, the rat model of liver fibrosis was confirmed by histopathological analysis and randomly grouped into the control, low, high dosage, and pentoxifylline (50 mg/kg) groups. These rats were orally administered BP (15 and 80 mg/kg) and olive oil daily for 15 and 30 d, combined with TAA-injections, and the rats were subsequently sacrificed. By using H&E stain, macrophage tissue infiltration decreased in the BP treatment groups at 6th and 8th week (activity score 1–2), whereas the olive oil group continued to exhibit substantial cell infiltration (activity score 3–4) (**Figure 1**). Masson’s trichrome stain for fibrotic tissue showed almost no collagen accumulation in the BP treatment groups at weeks 6 and 8 (fibrosis grade 1–2), whereas the olive oil group still exhibited a significant amount of collagen accumulation (fibrosis grade 2–3) (**Figure 1C**). The BP-treated groups exhibited significantly reduced AST and ALT levels, as shown in **Figure 1C** and 1 day, respectively. These data suggest that BP administration reduces both hepatic fibrosis and hepatic injury. BP reduced fibrosis earlier and more effectively than those treated with pentoxifylline.

### Butylidenephthalide Treatment Reduces Profibrogenic Proteins

**FIGURE 2:**
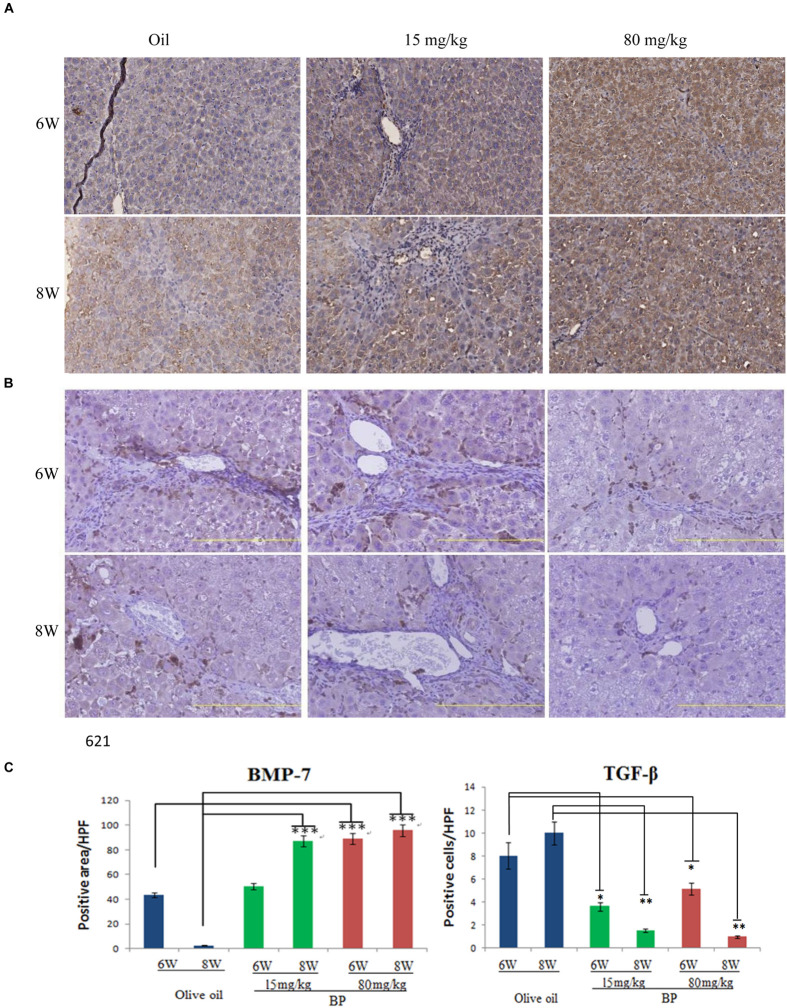
**Butylidenephthalide-decreased the expression of TGF-β, but increased BMP-7 dose-dependently in liver tissues.** The expression levels of **(A)** BMP-7 and **(B)** TGF-β in BP-treated livers was evaluated using immunohistochemical staining. **(C)** The intensity quantification of positive area was obtained by Aperio ePathology Software. Original magnification was 200×. (^∗^*p* < 0.05, ^∗∗^*p* < 0.01, ^∗∗∗^*p* < 0.001, Student’s *t*-test).

TGF-β is the most important profibrogenic cytokine, which induces EMT and causes fibrosis to occur. Thus, we analyzed the expression of TGF-β and its antagonist, BMP-7, by using immunohistochemistry. In the liver tissue, we observed the expression of BMP-7 in nearly all of the livers, and TGF-β was expressed only in the interlobular area (**Figures 2A,B**). The expression of BMP-7 and TGF-β was quantified, and was determined that the expression of BMP-7 was upregulated and that TGF-β was downregulated (**Figure 2C**). These data are consistent with the histopathological finding, suggesting that the expression state may be related to the anti-fibrosis effect.

### Butylidenephthalide Inhibits Epithelial–Mesenchymal Transition in Primary Rat Hepatic Stellate Cells

**FIGURE 3:**
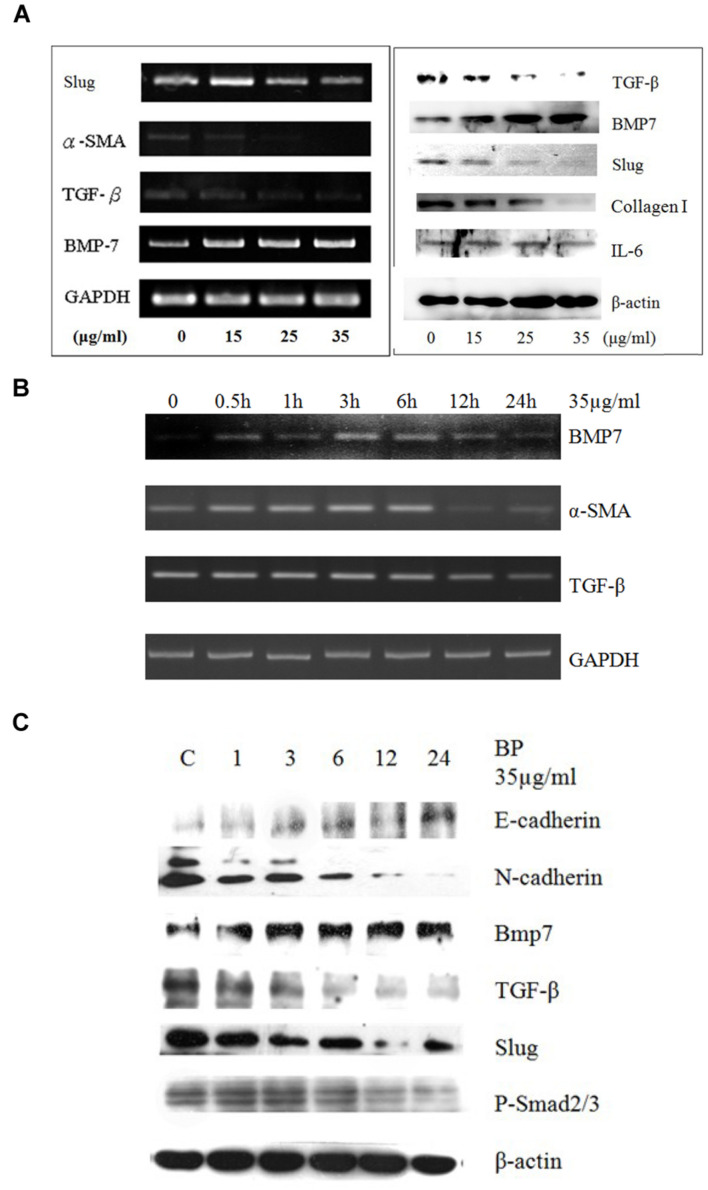
**Butylidenephthalide reduces EMT-related gene expression in dosage dependent manner *in vivo* and in cultured HSCs.** Primary HSCs treated with BP in **(A)** dosage-dependent manner using RT-PCR and western blot analysis. **(B)** Time-dependent study for the RNA expressions of BMP7, TGF-β, and α-SMA. **(C)** Western blot analysis to evaluated E- and N-cadherin. The GAPDH and β-actin content demonstrate that equal amounts of cDNA and protein had been loaded in each lane. The results represent three independent experiments.

Gene expression in cells treated with BP at 15 μg/mL, 25 μg/mL, or 35 μg/mL, or in a vehicle control, was analyzed using semiquantitive RT-PCR (**Figure 3A**). The expression of *BMP-7* increased in the BP-treated HSCs, whereas *TGF-β* and *α-SMA* expression decreased in a dose-dependent manner. In the time-dependent experiment, *BMP-7* expression increased between 6 and 12 h, while *TGF-β* expression decreased between 12 and 24 h, and lastly, the RNA expression of α-SMA decreased within 24 h. Compared with the protein expression which was determined using western blot analysis (**Figure 3C**), the expression of BMP-7 and TGF-β was affected at 24 h, and the phosphorylation of Smad 2/3 was reduced between 12 and 24 h. Studies have reported that the downstream genes of TGF-β in the EMT were induced through phospho-Smad (p-Smad) 2/3 and slug; the EMT pathway was induced by BMP-7 and through E-cadherin to reduce slug. BMP-7 was upregulated by BP at 6 h, and the expression of TGF-β and slug decreased; previous studies have obtained similar results indicating that slug is regulated by TGF-β and serves as the key regulator in the EMT. In addition, TGF-β-dependent p-Smad 2/3 was reduced by BP at 12 h, which was later than the time in which TGF-β was affected, suggesting that the EMT genes were regulated through p-Smad 2/3, a downstream signal conductor of TGF-β. Furthermore, a study reported that BMP-7 reverses the decrease of E-cadherin induced by TGF-β, suggesting that BP inhibits the EMT pathways through BMP-7 and TGF-β.

### Bone Morphogenetic Protein 7 Knockdown Blocks Butylidenephthalide-Inhibited Epithelial–Mesenchymal Transition Genes

**FIGURE 4:**
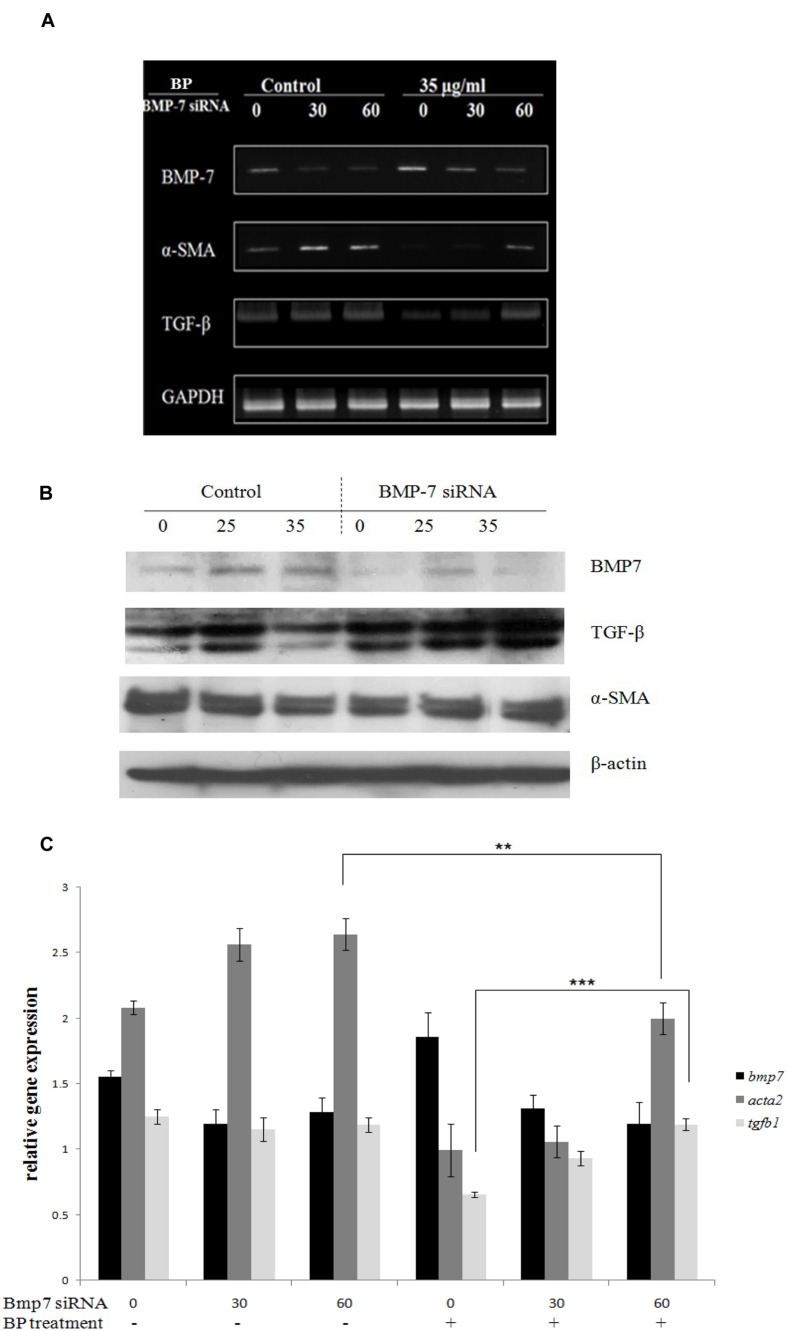
**BMP-7 knock down recovered α-SMA and TGF-β expression.** BP-induced BMP-7 secretion and BMP-7 knock down would reverse α-SMA expression. BMP-7 knock down by BMP-7 siRNA also reduced the secretion of BMP-7. BMP-7 knock down and followed BP treatment for 24 h, using **(A)** RT-PCR analysis for TGF-β, α-SMA, and BMP-7 expression. Further evidence by using **(B)** Western blot and **(C)** qRT-PCR to evaluate relative expressions. The relative expression values were calculated with standard curve by StepOne software. (^∗∗^*p* < 0.01, ^∗∗∗^*p* < 0.001, Student’s *t*-test).

Based on the *in vitro* data, we hypothesized that BP-induced BMP-7 inhibits the development of fibrosis in a TAA-treated rat liver. To obtain additional evidence, we examined BP-suppressed EMT in BMP-7-silenced HSCs by using siRNA transfection. Accordingly, BMP-7 siRNA (30 or 60 pmol) was added to 35-mm dishes containing HSC-T6 cells. The cells were treated with 35 μg/mL of BP or a vehicle control for 12 h, and mRNA expression was analyzed using RT-PCR (**Figure 4A**) and quantitative PCR in three independent transfections (**Figure 4C**). BMP-7 knockdown enabled the successful recovery of TGF-β expression after BP treatment, but α-SMA expression only slightly recovered, indicating that BP targets BMP-7 and inhibits TGF-β. These results were further confirmed by western blot analysis (**Figure 4B**).

### BP Suppresses Liver Fibrosis in Zebrafish by Regulating Bone Morphogenetic Protein 7

**FIGURE 5:**
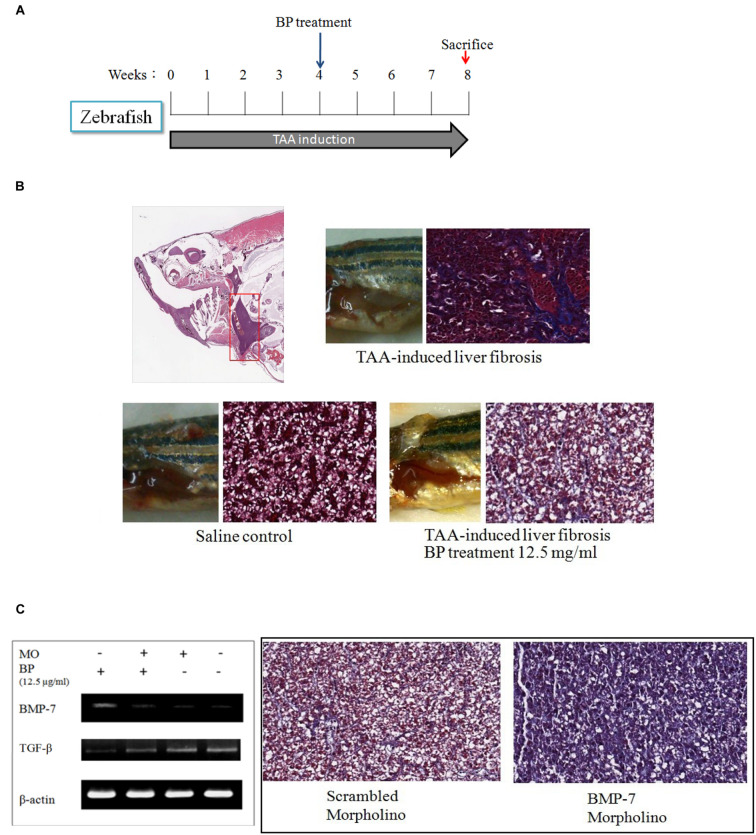
**TAA-induced liver fibrosis in zebra fish.** **(A)** Intraperitoneal injection of TAA (300 mg/kg) three times in a week. Liver tissues were then stained with Masson’s trichrom stain. **(B)** The collagen content in liver was indicated in blue color. The upper showed TAA-induced liver fibrosis; the lower left showed a normal liver, and lower right showed BP treated TAA-induced liver. **(C)** TAA-induced zebrafish was injected BMP-7 morpholino (MO) intravenously, and inhibited BP-induced BMP-7 expression (left). Two samples were treated with TAA, BP, and combined with scrambled-morpholino or BMP-7-MO treatment (iv).

To demonstrate that BP suppressed liver fibrosis by regulating BMP-7, we first developed TAA-induced liver fibrosis in zebrafish (**Figure 5A**). The TAA-induced liver fibrosis was obtained by performing intraperitoneal injections three times a week (300 mg/kg) and with the treatment of BP in the water of the aquarium. Fish liver was stained with Masson’s, which indicated that TAA-induces fibrosis and it was also ameliorated by BP (**Figure 5B**). *BMP-7* knockdown was treated by using morpholino (MO) and its expression analyzed by RT-PCR. The BP-reduced liver fibrosis was abolished by *BMP-7* knockdown (**Figure 5C**), suggesting that BP suppressed TAA induced liver fibrosis through BMP-7 in zebrafish.

### Butylidenephthalide Improves Liver Function and Proliferation

**FIGURE 6:**
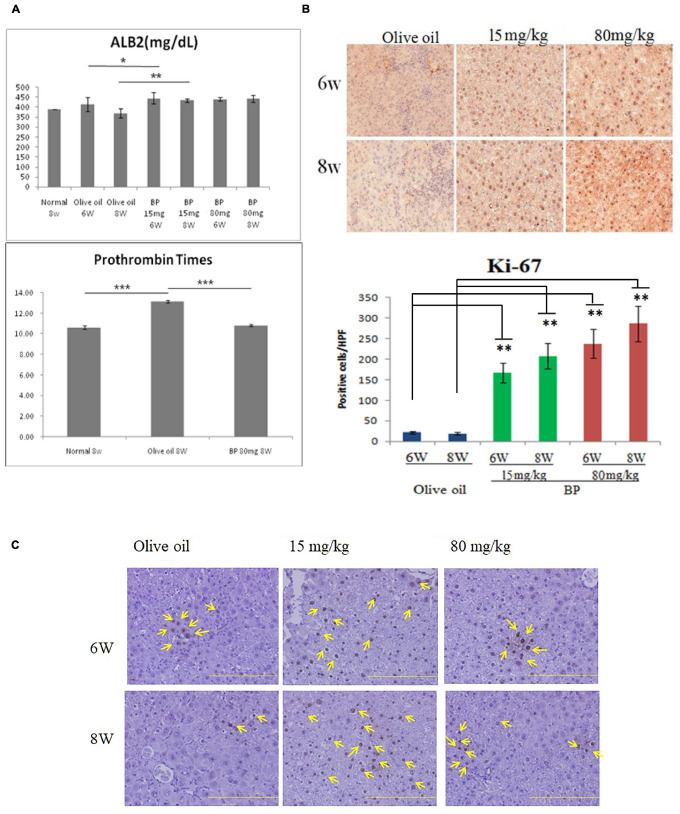
**Butylidenephthalide treatment improved liver function and proliferation.** Serum albumin level and prothrombin times evaluate liver functions in chronic liver fibrosis model. **(A)** Serum albumin content in 6 and 8 week rats treated with olive oil and BP. The lower graph showed that Prothrombin times comparison of normal, olive oil, and BP treatment. **(B)** Ki-67 expression evaluated by IHC and intensity quantification between treatments. **(C)** Oval cell specific marker OV-6 staining by IHC. (^∗^*p* < 0.05, ^∗∗^*p* < 0.01, ^∗∗∗^*p* < 0.001, Student’s *t*-test).

The blood test for albumin and clotting time were analyzed to evaluate liver functions, since albumin and clotting factors are synthesized by the liver. As shown in **Figure 6A**, serum albumin significantly increased in BP treatment groups. Shortened prothrombin time indicates that the blood clotting ability was enhanced between the control group and the BP treatment group (**Figure 6A**, bottom). Liver cell regeneration was revealed by analyzing the proliferation marker Ki-67 (**Figure 6B**). Furthermore, we analyzed the number of oval cells which differentiated into hepatocytes (**Figure 6C**). Livers administered with BP upregulated in their expression of Ki-67 which resulted in liver regeneration in chronically damaged liver.

### Butylidenephthalide Induces Matrix-Metalloproteinase Expression and Activation

**FIGURE 7:**
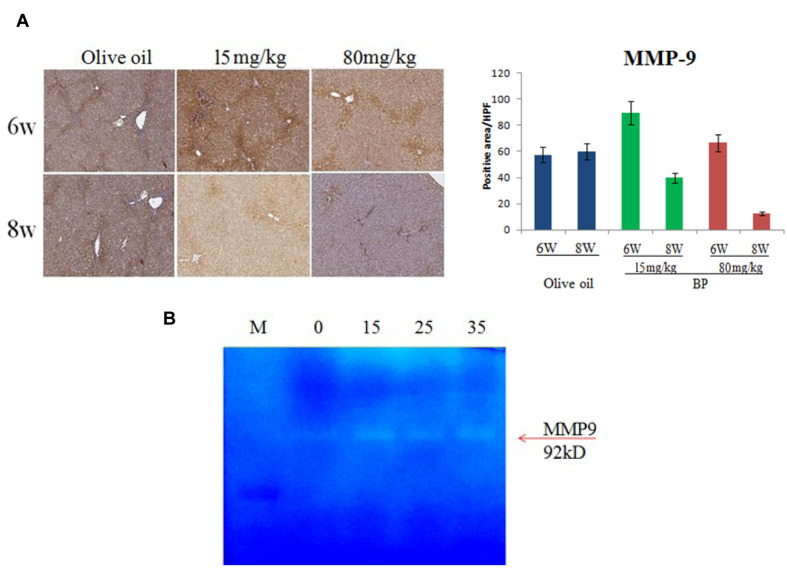
**Butylidenephthalide treatment induces MMP9 expression and activity.** MMP expression of fibrotic rat livers was indicated by IHC, with 6th, 8th week treatment with BP or olive oil as control **(A)**. *In vitro* study for MMP activity was demonstrated by zymography assay and secretion of HSC-T6 cells **(B)**.

The ECM accumulation resulted in the progression of fibrosis. In the liver regeneration stage, the improved liver cells secreted matrix metalloproteinases, digest collagen, elastin, and gelatin. To determine if BP can improve the digestion of ECM, we used IHC to indicate MMP-9 expression in rat livers. In **Figure 7A**, MMP-9 expression was upregulated on the 6th week by BP, whereas the control group stayed at the same expression state. Furthermore, the activities of MMPs were examined by zymography experiments. In previous reports, HSC was considered as one of the MMP-secreted cells. Thus, we used HSC-T6 cell lines to test the activity of MMPs. In **Figure 7B**, BP induced MMP9 activity in a dose-dependent manner, which is indicated by the digested gelatin (white band).

## Discussion

In recent years, many compounds have been reported to have anti-fibrotic effects in liver cells of animal models, but few have been approved for clinical use. A combined therapy treatment of PTX and corticosteroids (CS) was thought to be good because of its immune reaction, pro-inflammatory reduction, and pro-fibrogenic cytokines reduction; CS alone has limited effects (Sidhu et al., 2012). Sidhu et al. (2012) suggest the PTX and CS combination therapy explains the importance of anti-inflammatory could be less effectiveness as the anti-fibrosis targets. Alternatively, not only do these drugs cause inflammation and fibrotic cytokines, but it also reduces collagen fiber digestion and cell regeneration; these play an important role in the fibrotic microenvironment of the liver. Our results support the potential for both effects to improve liver fibrosis. As shown in **Figures 1A–C** and **Table 1**, the immune response in the portal areas and the accumulation of collagen fiber were reduced by BP. Moreover, BP downregulated EMT in Hepatic stellate cell by reducing TGF-β expression mediated by BMP-7 (**Figures 2A,B**), which led to decreasing collagen secretion. Furthermore, liver stem cell of oval cell increased in proliferation by BP. As a result, our data showed that the serum albumin and prothrombin times of rats administered with BP returned to their normal level (**Figure 6A**). We further found that BP treatment induced MMP-9 expression and its enzyme activity (**Figures 7A,B**), facilitates ECM degradation in the liver. In summary, it can be concluded that BP caused multiple effects on the microenvironment of the liver which led to liver fibrosis and to the liver dysfunction.

**Table 1:** Metavir scoring for hepatic fibrosis and serum biochemistry.

	Activity		Fibrosis		AST		ALT	
				
	6 Week	8 Week	6 Week	8 Week	6 Week	8 Week	6 Week	8 Week
Olive oil	2.95 ± 0.58	3.46 ± 0.36	3.2 ± 0.55	3.8 ± 0.45	307.7 ± 113.7	301.4 ± 92.76	115.8 ± 42.78	113.1 ± 29.41
PTX 50 mg	2.7^b^ ± 0.15	3.31^b^ ± 0.5	2.0^a^ ± 0.43	2.83^b^ ± 0.41	215.8^a^ ± 44.02	180.8^b^ ± 42.32	91.6^c^ ± 19.85	76.74^c^ ± 10.43
BP 15 mg	2.23^a^ ± 0.36	1.98^c^ ± 0.4	1.83^c^ ± 0.41	1.98^c^ ± 0.45	147.0^c^ ± 32.19	160.7^b^ ± 11.33	78.4^c^ ± 28.48	84.18^c^ ± 18.22
BP 80 mg	1.85^c^ ± 0.92	1.55^c^ ± 0.09	1.25^c^ ± 0.5	1.17^c^ ± 0.45	131.5^c^ ± 21.18	95.7^c^ ± 15.19	78.77^c^ ± 16.23	101.9^c^ ± 31.97


*The serum levels of activity, fibrosis, AST, and ALT in TAA-induced rat. Metavir score indicates immune cells infiltration and tissue fibrosis by Masson’s trichrome and H&E stain, respectively. BP administration was performed daily starting from the 4th week, samples were collected on 6th and 8th week points. (*n* = 5). (^a^*p* < 0.05, ^b^p < 0.01, ^c^p < 0.001, Student’s t-test).*

In previous studies, renal and hepatic fibrosis models have been shown to arise to about 40% of α-SMA–positive, collagen-secreting myofibroblasts (Xu et al., 2014) from the differentiation progenitors via EMT (Wynn and Ramalingam, 2012). EMT inducers like TGF-β and BMP-7 can be targeted for therapy; hence, we considered BP may reduce EMT, leading to the improvement in liver fibrosis. In our time-dependent study (**Figure 3C**), the protein expression of BMP-7 was upregulated at an early stage (1 h after BP treatment), in contrast to TGF-β, which decreased at the latter stage (12 h) of BP treatment. Also, the RNA expression of *bmp7* was induced at 0.5 to 1 h as shown in **Figure 3B**. These data reminds us that BP induced BMP-7 can regulate the expression of TGF-β; thereby, reducing liver fibrosis. To identify the relationship between BMP-7 and TGF-β, we knocked down the expression of BMP-7 by using siRNA and treated cells with BP for 24 h. We observed that the downregulated expression of TGF-β was partially recovered in BMP-7 knockdown cells, leading to an increase in EMT, which has been reported to promote liver fibrosis. Furthermore, to test whether BP reduces fibrosis in a BMP-7 deficient liver, an *in vivo* study of a zebrafish liver fibrosis model was conducted by administering TAA treatment (Rekha et al., 2008). We observed that BP did not reduce fibrosis in BMP-7 silenced fish (**Figure 5C**), suggesting that BMP-7 could be the upstream regulator of TGF-β and EMT genes. These results connect correlate? with previous studies on liver and renal fibrosis (Zeisberg et al., 2003; Kinoshita et al., 2007; Yang et al., 2012), suggesting that BMP-7 is one of the possible mechanisms in reversing liver fibrosis by counteracting TGF-β and EMT (Zeisberg et al., 2003).

In previous studies, MMP-9 (gelatinase B) was produced by Kupffer cells, Hepatocytes, and HSCs (Knittel et al., 1999b). As shown in **Figure 6A**, MMP-9 expression upregulated in 6w by BP treatment, and it has a positive stain in the portal area. Moreover, our results showed an increasing activity of MMP9 in the cultured HSC cells (**Figure 7B**). Hence, we hypothesized that MMP-9 secretion was induced in HSC by BP, and then the ECM was digested in the fibrotic livers. Unexpectedly, the MMP-9 expression was also found in the olive oil treatment, which is the solvent of BP used as the control of therapy. However, the Masson’s stain indicates the area of fibrotic scarring, which also expresses the MMP-9. Besides, previous reports have revealed that the withdraw of TGF-β will enhance ECM degradation and MMP activity (Arendt et al., 2005). In our results, BP induced the secretion of active MMP-9 on 15 μg/ml (**Figure 7B**); whereas, the dosage of reduction of TGF-β was about 25∼35 μg/ml, suggesting that TGF-β reduction is actually necessary in MMP-9-dependent ECM digestion. Furthermore, BP treatment was dismissed in BMP7 deficient zebrafish, which displayed collagen fibers in Masson’s stain. To summarize, BP not only induced activity of MMP, but also reduced TGF-β to facilitate metalloproteinase digestion. These findings suggest that BP regulated the microenvironment in order for the liver fibrosis to improve.

In summary, we demonstrated that BP reduced EMT by regulating BMP-7 in order to ameliorate liver fibrosis. The effect of MET was triggered by BMP-7 and inhibited by TGF-β, and thus prevented the activation of HSC and α-SMA expression. In addition, BP induced liver regeneration and enabled the recovery of liver function. Third, BP induced MMP-9 expression and fibrinolysis in the liver and reduced previously accumulated collagen. These data suggest that BP has the potential to effectively treat liver fibrosis and target the liver microenvironment for its antifibrotic effects.

## Author Contributions

H-MC wrote the paper and experiment. CL, S-YY, and M-HH help for the experiment. H-LS and S-ZL gave advises. H-JH and T-WC support funds.

## Conflict of Interest Statement

The authors declare that the research was conducted in the absence of any commercial or financial relationships that could be construed as a potential conflict of interest.

## References

[B1] Arendt E. Ueberham U. Bittner R. Gebhardt R. Ueberham E. (2005). Enhanced matrix degradation after withdrawal of TGF-beta1 triggers hepatocytes from apoptosis to proliferation and regeneration. *Cell Prolif* 38 287–299. 10.1111/j.1365-2184.2005.00350.x 16202037 PMC6495815

[B2] Avdesh A. Chen M. Martin-Iverson M. T. Mondal A. Ong D. Rainey-Smith S. (2012). Regular care and maintenance of a zebrafish (*Danio rerio*) laboratory: an introduction. *J. Vis. Exp.* 18:e4196. 10.3791/4196 PMC3916945 23183629

[B3] Bonacchi A. Petrai I. Defranco R. M. Lazzeri E. Annunziato F. Efsen E. (2003). The chemokine CCL21 modulates lymphocyte recruitment and fibrosis in chronic hepatitis C. *Gastroenterology* 125 1060–1076. 10.1016/S0016-5085(03)01194-6 14517790

[B4] Chan S. S. Jones R. L. Lin G. (2009). Synergistic interaction between the Ligusticum chuanxiong constituent butylidenephthalide and the nitric oxide donor sodium nitroprusside in relaxing rat isolated aorta. *J. Ethnopharmacol.* 122 308–312. 10.1016/j.jep.2009.01.002 19186210

[B5] Choi S. S. Diehl A. M. (2009). Epithelial-to-mesenchymal transitions in the liver. *Hepatology* 50 2007–2013. 10.1002/hep.23196 19824076 PMC2787916

[B6] Duarte S. Baber J. Fujii T. Coito A. J. (2015). Matrix metalloproteinases in liver injury, repair and fibrosis. *Matrix Biol.* 46C, 147–156. 10.1016/j.matbio.2015.01.004 PMC4495728 25599939

[B7] Fan H. C. Lin S. Z. Harn H. J. (2015). Targeting epithelial-mesenchymal transition phenotype for gastro-intestinal cancer. *Curr. Pharm. Des.* 21 2942–2955. 10.2174/1381612821666150514103513 26004417

[B8] Gunther U. Schuppan D. Bauer M. Matthes H. Stallmach A. Schmitt-Graff A. (1999). Fibrogenesis and fibrolysis in collagenous colitis. Patterns of procollagen types I and IV, matrix-metalloproteinase-1 and -13, and TIMP-1 gene expression. *Am. J. Pathol.* 155 493–503. 10433942 10.1016/S0002-9440(10)65145-0 PMC1866842

[B9] Harn H. J. Lin S. Z. Hung S. H. Subeq Y. M. Li Y. S. Syu W. S. (2012). Adipose-derived stem cells can abrogate chemical-induced liver fibrosis and facilitate recovery of liver function. *Cell Transplant.* 21 2753–2764. 10.3727/096368912X652959 22776464

[B10] Harn H. J. Lin S. Z. Lin P. C. Liu C. Y. Liu P. Y. Chang L. F. (2011). Local interstitial delivery of z-butylidenephthalide by polymer wafers against malignant human gliomas. *Neuro Oncol.* 13 635–648. 10.1093/neuonc/nor021 21565841 PMC3107093

[B11] Henderson N. C. Arnold T. D. Katamura Y. Giacomini M. M. Rodriguez J. D. Mccarty J. H. (2013). Targeting of alphav integrin identifies a core molecular pathway that regulates fibrosis in several organs. *Nat. Med.* 19 1617–1624. 10.1038/nm.3282 24216753 PMC3855865

[B12] Hindley C. J. Mastrogiovanni G. Huch M. (2014). The plastic liver: differentiated cells, stem cells, every cell? *J. Clin. Invest.* 124 5099–5102. 10.1172/JCI78372 25401467 PMC4348964

[B13] Kawada N. Tran-Thi T. A. Klein H. Decker K. (1993). The contraction of hepatic stellate (Ito) cells stimulated with vasoactive substances. Possible involvement of endothelin 1 and nitric oxide in the regulation of the sinusoidal tonus. *Eur. J. Biochem.* 213 815–823. 10.1111/j.1432-1033.1993.tb17824.x 7682947

[B14] Kendall R. T. Feghali-Bostwick C. A. (2014). Fibroblasts in fibrosis: novel roles and mediators. *Front. Pharmacol.* 5:123. 10.3389/fphar.2014.00123 PMC4034148 24904424

[B15] Kinoshita K. Iimuro Y. Otogawa K. Saika S. Inagaki Y. Nakajima Y. (2007). Adenovirus-mediated expression of BMP-7 suppresses the development of liver fibrosis in rats. *Gut* 56 706–714. 10.1136/gut.2006.092460 17127702 PMC1942155

[B16] Knittel T. Dinter C. Kobold D. Neubauer K. Mehde M. Eichhorst S. (1999a). Expression and regulation of cell adhesion molecules by hepatic stellate cells (HSC) of rat liver: involvement of HSC in recruitment of inflammatory cells during hepatic tissue repair. *Am. J. Pathol.* 154 153–167. 10.1016/S0002-9440(10)65262-5 9916930 PMC1853435

[B17] Knittel T. Mehde M. Kobold D. Saile B. Dinter C. Ramadori G. (1999b). Expression patterns of matrix metalloproteinases and their inhibitors in parenchymal and non-parenchymal cells of rat liver: regulation by TNF-alpha and TGF-beta1. *J. Hepatol.* 30 48–60. 10.1016/S0168-8278(99)80007-5 9927150

[B18] Lao S. C. Li S. P. Kan K. K. W. Li P. Wan J. B. Wang Y. T. (2004). Identification and quantification of 13 components in Angelica sinensis (Danggui) by gas chromatography-mass spectrometry coupled with pressurized liquid extraction. *Anal. Chim. Acta* 526 131–137. 10.1016/j.aca.2004.09.050

[B19] Lee T. F. Lin Y. L. Huang Y. T. (2007). Studies on antiproliferative effects of phthalides from *Ligusticum chuanxiong* in hepatic stellate cells. *Planta Med.* 73 527–534. 10.1055/s-2007-981520 17520522

[B20] Liu W. S. Lin P. C. Chang L. F. Harn H. J. Shiuan D. Chiou T. W. (2011). Inhibitory effect of n-butylidenephthalide on neointimal hyperplasia in balloon injured rat carotid artery. *Phytother. Res.* 25 1494–1502. 10.1002/ptr.3377 21365711

[B21] Liu X. Hu H. Yin J. Q. (2006). Therapeutic strategies against TGF-beta signaling pathway in hepatic fibrosis. *Liver Int.* 26 8–22. 10.1111/j.1478-3231.2005.01192.x 16420505

[B22] Pugach E. K. Li P. White R. Zon L. (2009). Retro-orbital injection in adult zebrafish. *J. Vis. Exp.* 7:1645. 10.3791/1645 PMC3149973 19997061

[B23] Rekha R. D. Amali A. A. Her G. M. Yeh Y. H. Gong H. Y. Hu S. Y. (2008). Thioacetamide accelerates steatohepatitis, cirrhosis and HCC by expressing HCV core protein in transgenic zebrafish *Danio rerio*. *Toxicology* 243 11–22. 10.1016/j.tox.2007.09.007 17997003

[B24] Satapathy S. K. Sakhuja P. Malhotra V. Sharma B. C. Sarin S. K. (2007). Beneficial effects of pentoxifylline on hepatic steatosis, fibrosis and necroinflammation in patients with non-alcoholic steatohepatitis. *J. Gastroenterol. Hepatol.* 22 634–638. 17444848 10.1111/j.1440-1746.2006.04756.x

[B25] Sidhu S. S. Goyal O. Singla P. Gupta D. Sood A. Chhina R. S. (2012). Corticosteroid plus pentoxifylline is not better than corticosteroid alone for improving survival in severe alcoholic hepatitis (COPE trial). *Dig. Dis. Sci.* 57 1664–1671. 10.1007/s10620-012-2097-4 22388710

[B26] Trautwein C. Friedman S. L. Schuppan D. Pinzani M. (2015). Hepatic fibrosis: Concept to treatment. *J. Hepatol.* 62 S15–S24. 10.1016/j.jhep.2015.02.039 25920084

[B27] Weiskirchen R. Meurer S. K. Gressner O. A. Herrmann J. Borkham-Kamphorst E. Gressner A. M. (2009). BMP-7 as antagonist of organ fibrosis. *Front. Biosci. (Landmark Ed.)* 14:4992–5012. 10.2741/3583 19482601

[B28] Wynn T. A. Ramalingam T. R. (2012). Mechanisms of fibrosis: therapeutic translation for fibrotic disease. *Nat. Med.* 18 1028–1040. 10.1038/nm.2807 22772564 PMC3405917

[B29] Xu J. Liu X. Koyama Y. Wang P. Lan T. Kim I. G. (2014). The types of hepatic myofibroblasts contributing to liver fibrosis of different etiologies. *Front. Pharmacol.* 5:167. 10.3389/fphar.2014.00167 PMC4105921 25100997

[B30] Yang T. Chen S. L. Lu X. J. Shen C. Y. Liu Y. Chen Y. P. (2012). Bone morphogenetic protein 7 suppresses the progression of hepatic fibrosis and regulates the expression of gremlin and transforming growth factor beta1. *Mol. Med. Rep.* 6 246–252. 10.3892/mmr.2012.892 22552821

[B31] Yen S. Y. Chen S. R. Hsieh J. Li Y. S. Chuang S. E. Chuang H. M. (2015). Biodegradable interstitial release polymer loading a novel small molecule targeting Axl receptor tyrosine kinase and reducing brain tumour migration and invasion. *Oncogene* 10.1038/onc.2015.277 [Epub ahead of print]. PMC4855077 26257061

[B32] Zein C. O. Yerian L. M. Gogate P. Lopez R. Kirwan J. P. Feldstein A. E. (2011). Pentoxifylline improves nonalcoholic steatohepatitis: a randomized placebo-controlled trial. *Hepatology* 54 1610–1619. 10.1002/hep.24544 21748765 PMC3205292

[B33] Zeisberg M. Hanai J. Sugimoto H. Mammoto T. Charytan D. Strutz F. (2003). BMP-7 counteracts TGF-beta1-induced epithelial-to-mesenchymal transition and reverses chronic renal injury. *Nat. Med.* 9 964–968. 10.1038/nm888 12808448

