# Correction: The role of butylidenephthalide in targeting the microenvironment which contributes to liver fibrosis amelioration

**DOI:** 10.3389/fphar.2026.1909286

**Published:** 2026-07-21

**Authors:** Hong-Meng Chuang, Hong-Lin Su, Chien Li, Shinn-Zong Lin, Ssu-Yin Yen, Mao-Hsuan Huang, Li-Ing Ho, Tzyy-Wen Chiou, Horng-Jyh Harn

**Affiliations:** 1 Agricultural Biotechnology Center, Department of Life Sciences, National Chung Hsing University, Taichung, Taiwan; 2 Department of Life Science and Graduate Institute of Biotechnology, National Dong Hwa University, Hualien, Taiwan; 3 Center for Neuropsychiatry, China Medical University Hospital, Taichung, Taiwan; 4 Graduate Institute of Immunology, China Medical University, Taichung, Taiwan; 5 Division of Respiratory Therapy, Department of Chest Medicine, Taipei Veterans General Hospital, Taipei, Taiwan; 6 Department of Pathology, China Medical University Hospital, Taichung, Taiwan; 7 Department of Medicine, China Medical University, Taichung, Taiwan

**Keywords:** liver fibrosis, epithelial-messenchymal transition, hepatic stellate cells, regeneration, microenvironment

There was a mistake in Figure 6C as published. The image representing the Olive oil (vehicle control) group at 8 weeks was inadvertently duplicated from the image used for the Olive oil group at 6 weeks. This error occurred during the manuscript preparation process when images were being arranged in Microsoft Word, resulting in the same micrograph appearing in both the 6-week and 8-week positions of the Olive oil group in Figure 6C. The corrected Figure 6C appears below.

**FIGURE 6:**
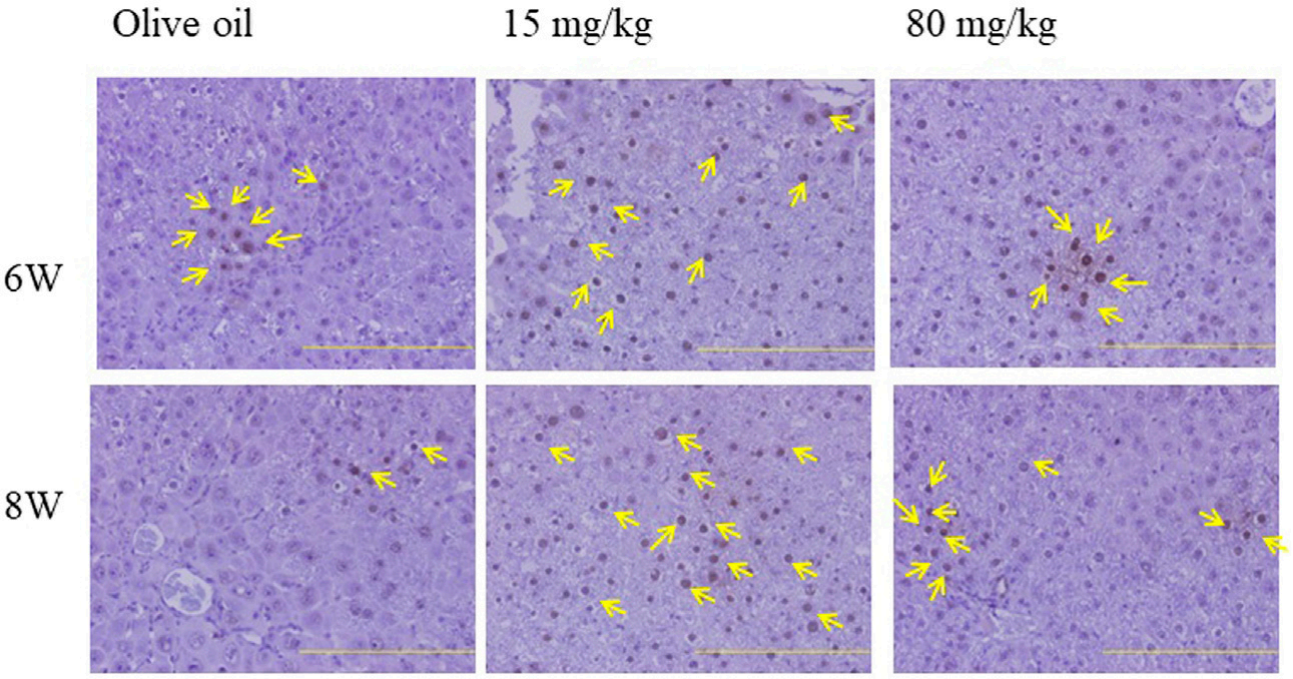
Butylidenephthalide treatment improved liver function and proliferation. Serum albumin level and prothrombin times evaluate liver functions in chronic liver fibrosis model. **(A)** Serum albumin content in 6 and 8 weeks rats treated with olive oil and BP. The lower graph showed that Prothrombin times comparison of normal, olive oil, and BP treatment. **(B)** Ki-67 expression evaluated by IHC and intensity quantification between treatments. **(C)** Oval cell specific marker OV-6 staining by IHC. (*p < 0.05, **p < 0.01, ***p < 0.001, Student’s t-test).

The original article has been updated.

